# Myocardial infarction following held antiplatelet therapy for lumbar radiofrequency neurotomy: A letter to the editor

**DOI:** 10.1016/j.inpm.2022.100171

**Published:** 2022-12-15

**Authors:** William Galbraith, Samir Khan, James E. Gardner, Byron Schneider

**Affiliations:** Department of Physical Medicine and Rehabilitation, Vanderbilt University Medical Center, Nashville, TN, USA

## Abstract

**Objectives:**

Describe a catastrophic complication of holding antiplatelet therapy (APT) prior to lumbar radiofrequency neurotomy (RFN).

**Setting:**

Bilateral L4-5 and L5-S1 RFN was performed 72 hours after the patient was instructed to hold APT.

**Discussion:**

Several hours after the lumbar RFN, the patient experienced substernal chest pain unresponsive to oral nitroglycerin. He presented to a local emergency department and was found to have ST-elevated myocardial infarction (STEMI), for which he underwent emergent cardiac catheterization. Almost all current literature and guidelines recommend continuing APT throughout the timeframe surrounding lumbar RFN yet the practice of holding these medications continues to be utilized by physicians.

**Conclusion:**

APT should not be discontinued in patients undergoing lumbar RFN due to the increased possibility of thromboembolic events such as a myocardial infarction.

Dear Editor,

Atherosclerosis is an arterial disease that can lead to cardiovascular disease (CVD), which continues to progress over time resulting in coronary artery disease (CAD) [1]. The prevalence and incidence of CVD is incredibly high and is the leading cause of death in the US [2]. Antiplatelet therapy (APT) is a mainstay therapy for the prevention and treatment of CVD, and particularly for CAD [3]. Patients with CVD and CAD are not immune to low back pain and often require the benefits that can be obtained from an interventional spine procedure. Lumbar RFN is a relatively safe procedure and-with proper screening and precise execution [4]- can have a high degree of efficacy as a treatment option for axial low back pain secondary to lumbar zygapophyseal joint dysfunction.

Recommendation and guideline are consistent throughout medical organizations regarding the risks and benefits of holding versus continuing antiplatelet medications prior to interventional spine procedures targeting zygapophyseal (facet) joint pain. The incidence of hemorrhagic complication following facet targeted intervention is remarkably low, with no published cases of clinically serious bleeding following the procedure [5].

In 2018 the American Society of Regional Anesthesia and Pain Medicine (ASRA) along with the American Academy of Pain Medicine (AAPM) and several other interventional pain societies released recommendations in the 2nd edition of Interventional Spine and Pain Procedures in Patients on Antiplatelet and Anticoagulant Medication in which they re-classified lumbar facet interventions from an intermediate risk to a low-risk procedure (Table 1). Within these guidelines they state that many, if not most, low-risk procedures can be safely performed without discontinuing P2Y12 inhibitors and that aspirin therapy not be interrupted for any low-risk interventions [[6], [7]].

**Table 1:** Pain procedure classification according to the potential risk for serious bleed; 2nd edition of Interventional Spine and Pain Procedures [6].

High-Risk Procedures	Intermediate-Risk Procedures	Low-Risk Procedures
Spinal cord stimulation trial and implant Dorsal root ganglion stimulation Intrathecal catheter and pump implant Vertebral augmentation Percutaneous decompression laminotomy Epiduroscopy and epidural decompression	Interlaminar ESI Transforaminal ESI Cervical facet MBNB and RFN Intradiscal procedures Sympathetic blocks Trigeminal and sphenopalatine ganglia blocks	Peripheral nerve blocks Peripheral joints and musculoskeletal injections Trigger point injections including piriformis injection Sacroiliac joint injection and sacral lateral branch blocks Thoracic and lumbar facet MBNB and RFN Peripheral nerve stimulation trial and implant

In 2020, the Spine Intervention Society (SIS) released a statement that the current evidence strongly suggests the risk of cerebrovascular and cardiovascular complications caused by the cessation of APT outweigh the potential risks of significant hemorrhagic complications in patients who maintain APT therapy during lumbar RFN [8].

The American Society of Interventional Pain Physicians (ASIPP) guidelines released in 2019 state there is fair evidence suggesting that the risk of a thromboembolic event after the interruption of antiplatelet therapy in anticipation of interventional techniques is higher than the risk of epidural hematoma formation associated with the continuation of antiplatelet therapy preceding interventional procedures, including lumbar RFN [9].

We present a case in which a patient was instructed to discontinue his APT for 72 hours prior to a lumbar RFN procedure, shortly after which he sustained a STEMI requiring emergent cardiac catheterization and revascularization. Awareness of this devastating complication should help add to the literature supporting the continuation of APT surrounding lumbar RFN.

This 2019 case involves a 64-year-old male with type two diabetes and coronary artery disease (CAD) status post bypass graft and percutaneous coronary interventions on dual-antiplatelet therapy (DAPT) of prasugrel and aspirin. The patient presented with 6 months of bilateral axial lumbosacral pain without radicular features; physical exam was suspicious for facet-mediated etiology, with exam findings positive for facet-loading maneuvers. X-ray imaging showed multi-level spondylosis including zygopophyseal joint facet arthropathy without fracture or malalignment. The patient had a positive response to dual controlled bilateral medial branch blocks (MBB) targeting the L4-5 and L5-S1 zygopophyseal joints and thus proceeded to RFN. The patient's interventional physician instructed him to hold DAPT (aspirin and prasugrel) for 72 hours prior to RFN, as he had for the previous MBB, a decision that had been previously agreed upon by the patients cardiologist, who communicated their assent to the patient, prior to the first MBB. The providers reasoning for holding DAPT for 72 hours was not found in their clinical or procedural notes despite being in direct conflict with ASRA, SIS and other guidelines. The two MBB's and the RFN were all performed 2 weeks apart and DAPT was held 72 hours prior to each. The planned RFN procedure was performed without complications.

The patients care team placed him in the prone position, the skin over and surrounding the treatment area was cleaned and covered with sterile drapes. Over the L4, L5, and S1 superior articular process (SAP), the skin and subcutaneous tissue within the planned approach was anesthetized with 1% lidocaine. With intermittent fluoroscopy, the performing anesthesiologist guided the treatment needles to the interface of the SAP with its respective lateral mass. Needle localization was confirmed with AP (Fig. 1) and lateral (Fig. 2) radiographs. 2 ​cc of 1% lidocaine was injected at each site wherein a lesion was then created with a temperature of 80° Celsius for 90 seconds x2, and a third lesion at 80° for 60 seconds. Upon completion, the needles were withdrawn, the skin was cleaned, and sterile bandages were placed over the needle puncture sites.

**Fig. 1:**
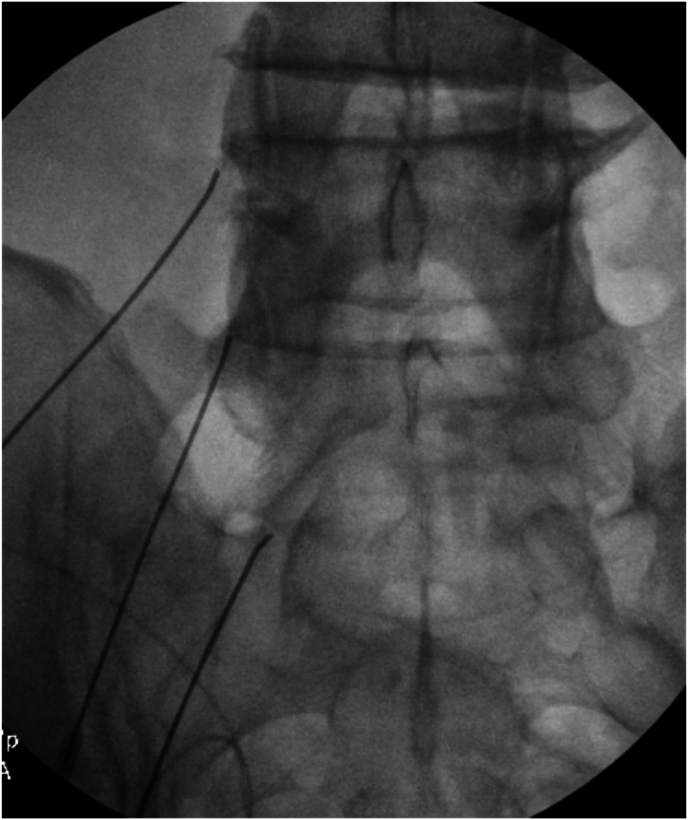
Fluoroscopic image, AP view of lumbosacral needle placement during RFN, targeting the left medial branch of L3 and L4 and dorsal ramus of L5. Imaging showing sacralization of L5 vertebra.

**Fig. 2:**
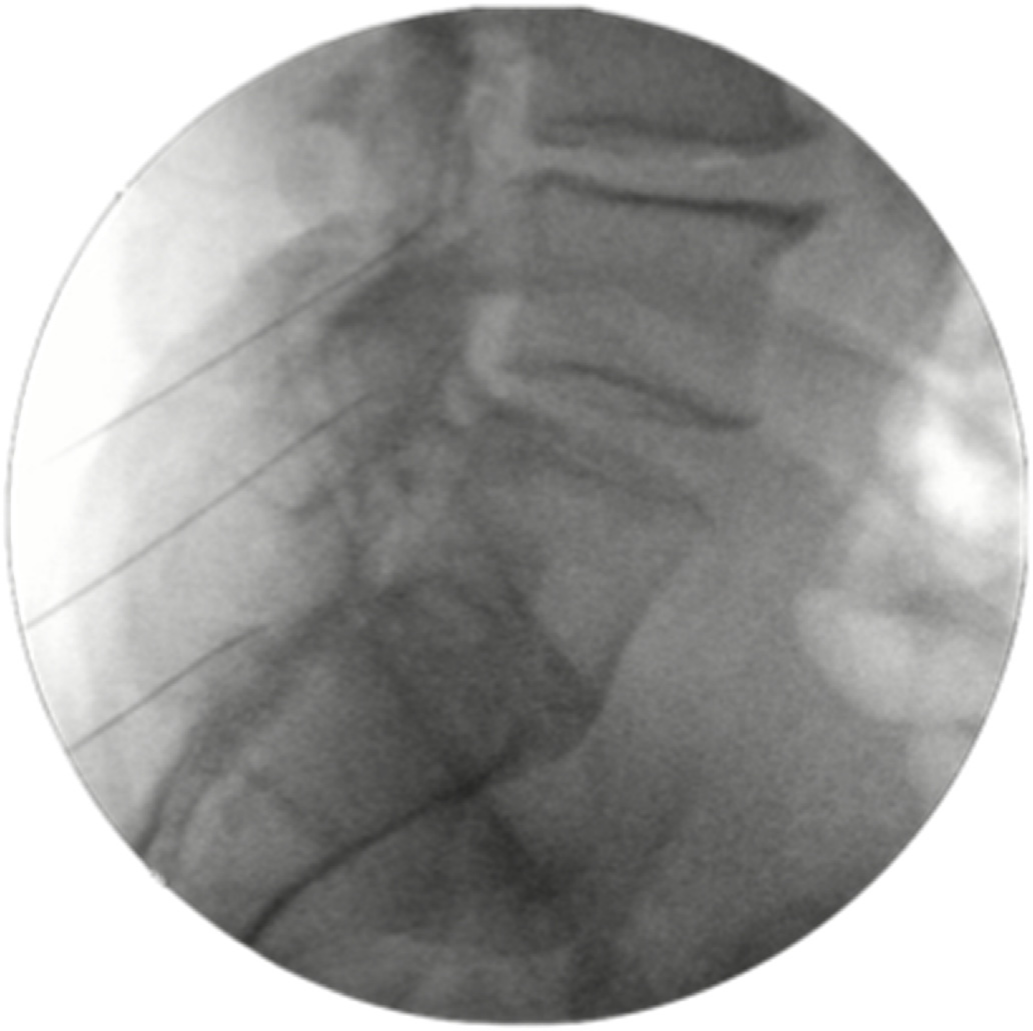
Fluoroscopic image, lateral view of lumbosacral needle placement during RFN.

At the time of post-procedural discharge, the patient was neurologically intact and otherwise in his normal state of health. Later that afternoon, while at home, he began experiencing substernal chest pain unresponsive to oral nitroglycerin. The patient immediately presented to his local emergency department where Electrocardiogram demonstrated ST elevation consistent with myocardial infarction. The patient was then taken for emergent cardiac catheterization which revealed occlusion of the left anterior descending artery requiring stenting for coronary revascularization.

Due to the close association of discontinuing DAPT for 72 hours and then sustaining a STEMI, his cardiology team maintained a high suspicion for this being the etiology of his thromboembolic event. Though holding DAPT may have been the cause or a contributed factor to the patient having an MI other cause cannot be ruled out due to the patient having known CAD and type 2 diabetes. There were no reports of the patient having elevated blood pressure or any other abnormal vital signs during or immediately after the procedure. The patient ultimately survived the ordeal but any potential sequala from the event is unknown due to the patient being lost to follow-up at the Pain clinic and changing cardiologist.

A systematic review of lumbar RFN procedures in patients on APT reported no bleeding complications [10]. And though the risk of thrombotic events due to holding anticoagulant and antiplatelet medications prior to spine procedures is still markedly low-estimated to be around 0.4%- the sequelae of such an event can be catastrophic [11]. Despite the current evidence and the presence of numerous guidelines from various medical associations and societies, there continues to be practice inconsistencies between individual physicians, for reasons that can only be speculated.

This case illustrates a severe cardiovascular thrombotic event following lumbar RFN for which APT was held. The potentially devastating complications detailed in this case add to the growing body of evidence supporting the current mainstream recommendations to continue APT/DAPT prior to facet targeted interventions including RFN. This also serve to further highlight the need for these practices to become standard of care amongst all physicians as well as the need for ongoing dissemination of this information to both pain and spine physicians as well as other medical professionals included in patient care including the NP and PA's who work in pain and spine clinics and the cardiologist and PCP's who assist in managing these medications.

## Declaration of competing interest

The authors declare that they have no known competing financial interests or personal relationships that could have appeared to influence the work reported in this paper.
